# The Financial Burden of Antiquated Laws: The Case of Massachusetts' Parental Involvement Law for Abortion

**DOI:** 10.1089/whr.2021.0002

**Published:** 2021-11-29

**Authors:** Allison L. Gilbert, Isabel R. Fulcher, Alischer A. Cottrill, Elizabeth Janiak

**Affiliations:** ^1^ Department of Obstetrics, Gynecology and Reproductive Biology, Brigham and Women's Hospital, Boston, Massachusetts, USA.; ^2^ Southwestern Women's Surgery Center, Dallas, Texas, USA.; ^3^ Department of Global Health and Social Medicine, Harvard Medical School, Boston, Massachusetts, USA.; ^4^ Planned Parenthood League of Massachusetts, Boston, Massachusetts, USA.

**Keywords:** parental involvement laws, judicial bypass, cost of judicial bypass, minors, abortion

## Abstract

***Background:*** A majority of U.S. states enforce parental involvement laws that require minors seeking abortion to obtain parental consent, or else obtain judicial bypass through the court system. Although such laws are widespread, the financial cost of their enforcement has yet to be documented.

***Methods:*** We used data from a retrospective observational cohort study among adolescents (aged ≤17 years old) who sought abortion services at Planned Parenthood League of Massachusetts (PPLM) between 2010 and 2016. We assessed the direct financial burden of judicial bypass among 449 minors accounting for direct public legal costs, private professional costs, cost of lost school, and cost to the young person.

***Results:*** The total added cost of judicial bypass in our cohort amounted to $374,982.04 (median cost of $705.14 per abortion). The direct out-of-pocket cost amounted to $84,370.23 ($179.89 per abortion). The majority of this cost was due to increased average procedure costs solely due to delays in care incurred by judicial bypass (range $0 to $5,200.50). In total, 74% of minors in our cohort were insured through Medicaid at the time of their abortion. Additional out-of-pocket costs for bypass were 20.2% of their household's maximum monthly income.

***Conclusions:*** These analyses show that judicial bypass as a function of parental involvement laws correlates with increased costs to individual minors and to the public, with the heaviest burden placed on minors of low socioeconomic status.

## Introduction

Thirty-seven states currently enforce parental involvement laws for legal minors obtaining abortion care.^1^ Among the oldest of these laws is Massachusetts' parental consent law, which requires the consent of at least one parent for all youth aged 17 years and below (excepting those who are married, widowed, or divorced), and which has been in effect since 1981.^2^ Legal challenges to Massachusetts' law have established the standard for constitutionality against which all state-level parental involvement laws have been judged.

According to the U.S. Supreme Court's *Bellotti vs. Baird* decisions, parental involvement laws are permitted so long as young people are able to bypass this requirement through an external process.^2,3^ In most states, this process takes the form of a judicial bypass, whereby a young person must petition a judge to be ruled mature enough to make the abortion decision on her own, without parental involvement.^3^

**FIG. 1.**
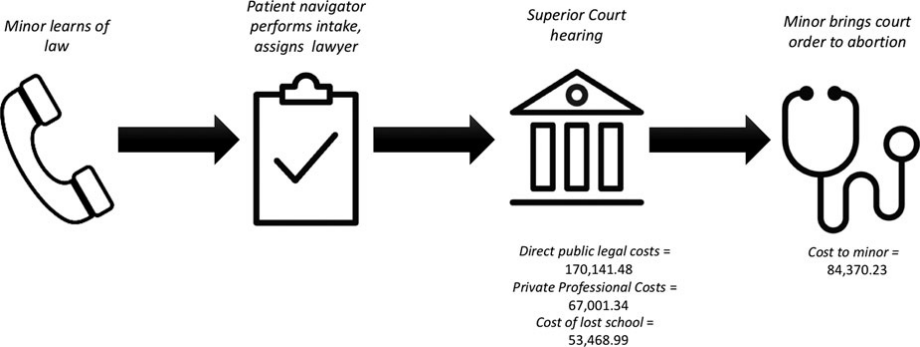
Judicial bypass process.

In Massachusetts, young people seeking judicial bypass can obtain free legal representation through a statewide care navigation program housed at Planned Parenthood League of Massachusetts (PPLM). Upon deciding to seek judicial bypass, a young person is connected to a care navigator who then assigns them to legal representation from a statewide panel of specially trained attorneys at no cost. Attorneys schedule a confidential hearing with a judge at a Massachusetts Superior Court and accompany their client throughout the process (Fig. 1). These hearings take place on weekdays during standard work and school hours.

Literature on judicial bypass is limited, but prior research has found the process to be associated with out-of-state travel for abortion,^4,5^ psychological distress among young people who have undergone the process, and clinically significant delays in abortion care.^3,6,7^ Our prior research demonstrated that Massachusetts' parental involvement law delays care for minors seeking abortion.^6^

Those who obtain judicial bypass have an average additional delay of 6.1 days compared with those with parental consent, but for one in five minors receiving bypass, this process takes 21 days or longer.^6^ Delays from parental involvement laws may result in costlier abortion procedures for some young people. In addition, there are direct costs associated with obtaining judicial bypass, such as missed schooling, lost wages, and transportation costs.

Although the physical and psychological consequences of undergoing bypass have begun to be illuminated in the research literature, the financial cost of compliance with these laws has yet to be documented. We set out to fill this gap using data from a cohort of minors who underwent judicial bypass in Massachusetts over a 5-year period.

## Methods

We used data from a retrospective observational cohort study among adolescents (aged ≤17 years) who sought abortion services at PPLM) clinics between 2010 and 2016. We identified 479 instances of minors who obtained a judicial bypass for their abortion procedure, of whom 10 (2.1%) were excluded as they were lost to follow-up after requesting an abortion. This resulted in a final sample size of 469 abortions corresponding to 449 minors, as some minors had multiple abortion procedures during the study period.

Our cohort included minors who received an abortion at PPLM, which had an institutional gestational age limit of 18 weeks and 6 days for abortions throughout the study period, as well as those who were referred from PPLM to an outside provider owing to a gestational age of 19 weeks or later.

Data were abstracted both from the PPLM medical record and from a patient referral database maintained by care navigation staff containing data of patient living situation, work, and school. Publicly available sources were used to determine cost for all items except procedure cost. For procedure cost, private facilities were queried for cost estimates at comparable gestational ages. Neither medical records nor patient-identifying information was used to obtain cost estimates. This study was approved by the Partners Human Research Committee.

For each abortion, we calculated the cost of the judicial bypass using an “ingredients-based” approach.^8,9^ We tabulated costs incurred by the public (*i.e.*, court proceedings), private entities (*i.e.*, attorney fees and case management), and the individual minor (*i.e.*, lost work wages, childcare, travel expenses, or the need for a more expensive procedure due to care delays), as well as the cost of missed school (Table 1). For expenses due to lost work or school hours, we considered both time spent in the 1-hour prehearing meeting and a full day spent in court.

**Table 1.** Estimated Average Costs Per Abortion Procedure

Cost item	Cost	Applicable population
Direct public legal costs^14^		
Court reporter salary	$58.74	All minors
Paralegal salary	$44.84	All minors
Judge salary	$94.20	All minors
Attorney fee billed to state	$165.00	All minors
Private professional costs^14^		
Additional attorney value	$81.24	All minors
Case management salary	$61.62	All minors
Cost of lost school		
Public^18^	$130.79^a^	90.5% of minors with abortions during school year^8^
Private^19^	$193.52^a^	9.5% of minors with abortions during school year^8^
Cost to minor		
Lost work^11^	$108.00	Minors with abortions during summer
Lost work^11^	$60.00	Minors with abortions during school year
Childcare^12^	$80.50	Minors with at least one prior birth
Travel^13^	$18.77	All minors
Additional procedure cost	$121.07	Minors with abortion delays due to bypass

^a^ Operationalized as an average cost of $136.75 to every abortion during school year.

Lost school was only included for minors who reported school enrollment at the time of their procedure and whose procedure took place during the school year (September through June, *n* = 391). We assumed that 90.5% of minors attended public school ($114.44 per day) and the remaining 9.5% attended private school ($169.35 per day).^10^ Based on these percentages, we calculated an average for each minor as it was impossible to identify which individuals were enrolled in public school versus private school. Lost work was only included for minors who were currently employed (*n* = 198).

For minors with a July or August abortion, we assumed 8 hours of lost work; for minors with an abortion during the school year, we assumed 4 hours of lost work ($12 per hour).^11^ The cost of daycare was only applicable to minors with at least one prior birth (*n* = 46) and was calculated only for one day in court ($80.50 per day).^12^ For travel to court, we used the federal reimbursement rate for gas based on the roundtrip distance from the minor's ZIP code to the court they attended ($0.36 per kilometer).^13^

Salaries were calculated for court reporters ($39.16 per hour), paralegals ($29.89 per hour), and judges ($62.80 per hour) from median salaries reported by the Bureau of Labor Statistics.^14^ In Massachusetts for judicial bypass cases, lawyers only bill the state a nominal fee ($55.00 per hour) while their median salary is higher ($82.08 per hour). We used the cost billed to the state in the calculation of total public cost and considered the additional lost wages as a private cost. We also included the cost of a case manager and assumed 2 hours per minor ($30.81 per hour).

We assumed that each lawyer spent 3 hours total on the case, and that court reporters, paralegals, and judges each spent 1.5 hours during the minor's court proceedings. Time spent was estimated from expert consultation (Jamie Sabino, JD, personal communication, August 27, 2019). It was assumed that all minors had the same direct public legal costs and private professional costs ($505.64 each).

Owing to delays in care incurred by the judicial bypass process, some minors paid more for their abortion procedure. For these cases, we calculated the difference between expected procedure cost based on gestational age at point of first contact for the abortion procedure and actual procedure cost based on what the minor actually paid. For minors who received their procedure at PPLM, abortion costs were based on the current cost of each procedure at PPLM as of November 2019.

Of the 21 minors who had a procedure outside of PPLM, only 7 required outside care due to their gestational age being past the PPLM threshold of 18 weeks, 6 days after bypass. Of these, six had their procedure at a hospital in Massachusetts ($5,950.50 per procedure, cost quoted from hospital administrator). The remaining minor traveled out-of-state for her procedure (location known but redacted). For this minor, abortion costs were derived from the procedure cost at the clinic attended ($6,500.00), a one-night stay in a hotel ($107.00), and a roundtrip flight to the state where the clinic was located ($385.59).^15^

Some minors had missing values for cost variables: 34 were missing employment status, 18 travel cost, and 1 gestational age at procedure. To calculate a final cost for each minor, the missing values were assigned the average cost among all minors for the relevant expense. To ensure that his did not impact the accuracy of cost estimates, we also calculated best (and worst) case total costs by imputing the lowest (and highest) possible cost for each missing value. We report an overall total cost for the study population broken out by expense category, as well as the total cost for each abortion.

### Patient and public involvement

There was no involvement of patients or the public in the design of any aspect of this study.

## Results

The total cost of the 469 abortions provided to 449 minors through Massachusetts's judicial bypass system from 2010 to 2016 was $374,982.04 (best-worst case range: $373,433.61, $383,248.32) (Table 2). This amounts to a median additional cost of $705.14 per abortion ($701.98, $707.36). The cost of public court proceedings totaled $170,141.48 ($362.78 per abortion). This includes the nominal fee paid to lawyers by the state, as well as the salaries of judges, court reporters, and paralegals. Other factors such as ancillary employee salaries and energy costs associated with the operation of a courtroom were not included.

**Table 2.** Estimated Cumulative Cost for Entire Cohort from Authors' Analysis

Cost item	Average cost	Lowest cost	Highest cost
Direct public legal costs			
Attorney fee billed to state	$77,385.00	—	—
Judge salary	$44,179.80	—	—
Court reporter salary	$27,549.06	—	—
Paralegal salary	$21,027.62	—	—
Private professional costs			
Additional attorney value	$38,101.56	—	—
Case manager salary	$28,899.78	—	—
Cost of lost school			
Public	$48,389.44	—	—
Private	$5,079.55	—	—
Cost to minor			
Lost work	$15,085.63	$13,992.00	$17,016.00
Childcare	$3,703.00	—	—
Travel	$8,801.54	$8,467.80	$12,458.51
Additional procedure cost	$56,780.07	$56,659.00	$59,459.00
Total	374,982.04	373,433.61	383,248.32

**FIG. 2.**
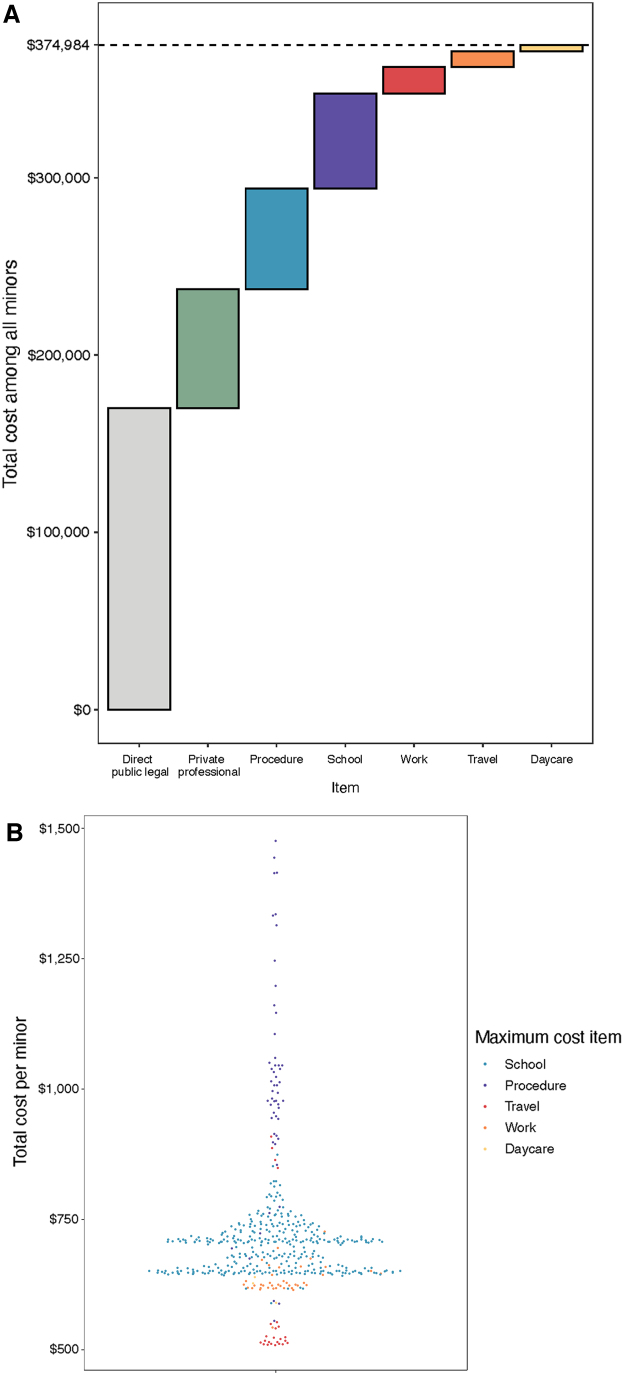
Costs of judicial bypass (*n* = 469). **(A)** Displays the contribution of each item to the total cost in the cohort. **(B)** Displays the total cost per minor in the cohort colored by maximum cost item (excluding state as all minors had the same value and *n* = 8 minors with costs >$4,000 due to procedure); each dot represents a minor.

The cost incurred by private entities totaled $67,001.34 ($142.86 per abortion). This cost includes the value of labor contributed by PPLM staff and attorneys working on a pro-bono basis. The average value of missed school totaled $53,468.99 ($114.01 per abortion). The vast majority of this amount could be considered public cost, as 90.5% of minors in Massachusetts are enrolled in public school.^10^ (Fig. 2A).

The additional cost paid directly out-of-pocket by each minor was $84,370.23 ($179.89 per abortion). The majority of this cost was due to increased procedure costs due to delays in care incurred by judicial bypass ($121.07 per abortion). The remaining individual cost was calculated from lost wages, daycare, and travel to and from the courthouse. (Fig. 2B).

## Discussion

Despite widespread enforcement of parental involvement laws for abortion care throughout the United States, denials of judicial bypass petitions remain rare.^16^ Nonetheless, Massachusetts—along with 36 other states—maintains antiquated laws that require minors who cannot obtain parental consent to undergo a long and costly court process. Judicial bypass has been shown to have a myriad of adverse effects on young people, including clinically significant delays in abortion care.^6^ Our analysis further demonstrates the impact of these laws by quantifying the high financial cost of their enforcement.

The financial burden of judicial bypass is especially stark for minors of low socioeconomic status. Among our cohort, 74% of minors were on Medicaid at the time of their abortion, indicating a household income of <$891 per month or $10,692 per year for a family of four.^17^ The additional out-of-pocket costs of abortion for a minor undergoing judicial bypass in our data set were on average $179.89, a significant proportion of their household's monthly income (20.2%).

These results should be interpreted within the context of several limitations. First, this study underestimates the total cost of judicial bypass by design, as we do not account for lost economic and educational productivity due to lost time for court proceedings. We relied on median salary information from the Bureau of Labor Statistics for each occupational code, which likely underestimates total wage costs. Second, we use costs associated with judicial bypass to estimate the financial burden of parental involvement laws, but we do not consider the costs incurred by minors who have parental consent.

The Massachusetts parental involvement law could also raise abortion-related costs for those with parental consent, since parents may need to miss work or obtain childcare to accompany their child to the abortion appointment, when some may have otherwise been accompanied by a friend, older sibling, or other adult relative. Furthermore, there is likely additional variation in cost between minors that was not captured by the demographic characteristics we considered. This study is only meant to provide a best estimate of cost based on the available information from outside sources.

Future studies that directly collect information on costs from abortion patients would be able to address these concerns. But perhaps most significantly, we are unable to quantify the cost to minors who choose not to seek an abortion because of these financial barriers. Despite these conservative estimates, judicial bypass clearly correlates with increased costs to individual minors and to the public at large, with a disproportionate burden placed on minors of low socioeconomic status.

**Key Messages**

- A majority of U.S. states require minors seeking abortion to obtain parental consent, or else obtain judicial bypass through the court system.
- Judicial bypass is associated with increased costs to individual minors and to the public.
- These laws disproportionately burden minors of low socioeconomic status.

## Abbreviation Used

PPLM: Planned Parenthood League of Massachusetts

## References

[1] Guttmacher Institute. State policies in brief: parental involvement in minors' abortions [Internet]. New York: Guttmacher Institute; 2020 May [cited May 22, 2020]. Available at: https://www.guttmacher.org/state-policy/explore/parental-involvement-minors-abortions Accessed May 22, 2020.

[2] Bellotti v. Baird, 443U.S. 622 (1979). Available at: https://supreme.justia.com/cases/federal/us/443/622/ Accessed July 1, 2020.

[3] Ehrlich JS. Who decides? The abortion rights of teens. Westport, CT: Prager, 2003.

[4] Cartoof VG, Klerman LV. Parental consent for abortion: Impact of the Massachusetts law. Am J Public Health 1986;76:397–400. 3953915 10.2105/ajph.76.4.397 PMC1646500

[5] Dennis A, Henshaw SK, Joyce TJ, Finer LB, Blanchard K. The impact of laws requiring parental involvement for abortion: A literature review. New York: Guttmacher Institute, 2009.

[6] Janiak E, Fulcher IR, Cottrill AA, et al. Massachusetts' parental consent law and procedural timing among adolescents undergoing abortion. Obstet Gynecol 2019;133:978–986. 30969206 10.1097/AOG.0000000000003190 PMC6485490

[7] Coleman-Minahan K, Stevenson AJ, Obront E, Hays S. Young women's experiences obtaining judicial bypass for abortion in Texas. J Adolesc Health 2019;64:20–25. 30197199 10.1016/j.jadohealth.2018.07.017 PMC7274206

[8] Johns B, Baltussen R, Hutubessy R. Programme costs in the economic evaluation of health interventions. Cost Eff Resour Alloc. 2003;1:1. 12773220 10.1186/1478-7547-1-1 PMC156020

[9] Lince-Deroche N, Constant D, Harries J, et al. The costs and cost effectiveness of providing second-trimester medical and surgical safe abortion services in Western Cape Province, South Africa. PLoS One 2018;13:e0197485. 29953434 10.1371/journal.pone.0197485 PMC6023192

[10] Student Enrollment and Indicators: Massachusetts Department of Education; 2019 [cited May 22, 2020]. Available at: http://profiles.doe.mass.edu/state_report/#Student%20Enrollment%20and%20Indicators Accessed August 16, 2019.

[11] State Mimimum Wage Laws: US Department of Labor; 2019 [cited May 22, 2020]. Available at: https://www.dol.gov/agencies/whd/minimum-wage/state#stateDetails Accessed September 19, 2019.

[12] Parents and the High Cost of Child Care: Child Care Aware of America; 2017 [cited May 22, 2020]. Available at: https://usa.childcareaware.org/wp-content/uploads/2017/12/2017_CCA_High_Cost_Report_FINAL.pdf Accessed June 18, 2019.

[13] IRS Issues Standard Mileage Rates for 2019: Internal Revenue Service; 2019 [cited May 22, 2020]. Available at: https://www.irs.gov/newsroom/irs-issues-standard-mileage-rates-for-2019 Accessed June 18, 2019.

[14] Occupational Employment Statistics: Bureau of Labor Statistics; 2019 [cited May 22, 2020]. Available at: https://www.bls.gov/oes/current/oes_71650.htm#23-0000 Accessed September 19, 2019.

[15] Statistics BoT. Average Domestic Airline Itinerary Fares; 2019 [cited May 22, 2020]. Available at: https://www.transtats.bts.gov/AverageFare/ Accessed January 31, 2020.

[16] Stevenson AJ, Coleman-Minahan K, Hays S. Denials of judicial bypass petitions for abortion in Texas before and after the 2016 bypass process change: 2001–2018. Am J Public Health 2020;110:351–353. 31944836 10.2105/AJPH.2019.305491 PMC7002964

[17] MassHealth Income Standards and Federal Poverty Guidelines; 2019 [cited May 22, 2020]. Available at: https://www.mass.gov/doc/2019-masshealth-income-standards-and-federal-poverty-guidelines/download Accessed January 31, 2020.

[18] Boston Public School Budget Basics: Boston Public Schools; 2019. Available at: https://www.bostonpublicschools.org/page/7499 Accessed August 16, 2019.

[19] Top Boston Private Schools: Private School Review; [cited June 2, 2020]. Available at: https://www.privateschoolreview.com/massachusetts/boston Accessed August 16, 2019.

